# Gaseous mediators: an updated review on the effects of helium beyond blowing up balloons

**DOI:** 10.1186/s40635-019-0288-4

**Published:** 2019-12-19

**Authors:** Nina C. Weber, Benedikt Preckel

**Affiliations:** Amsterdam University Medical Centers, location AMC, Meibergdreef 9, 1105 AZ Amsterdam, the Netherlands

**Keywords:** Noble gases, Helium, Organ protection, Conditioning, Airway obstruction, Ventilation

## Abstract

Noble gases, although supposed to be chemically inert, mediate numerous physiological and cellular effects, leading to protection against ischaemia-reperfusion injury in different organs. Clinically, the noble gas helium is used in treatment of airway obstruction and ventilation disorders in children and adults. In addition, studies from recent years in cells, isolated tissues, animals and finally humans show that helium has profound biological effects: helium applied before, during or after an ischaemic event reduced cellular damage, known as “organ conditioning”, in some tissue, e.g. the myocardium. Although extensive research has been performed, the exact molecular mechanisms behind these organ-protective effects of helium are yet not completely understood. In addition, there are significant differences of protective effects in different organs and animal models. A translation of experimental findings to the clinical situation has yet not been shown.

## Background

When we ask our colleagues what they could do with helium, responses are mostly to blow up balloons for their children, to produce a funny voice, or to improve diving efficiency [1]. However, although helium is supposed to be chemically inert, it mediates numerous cellular effects, leading to physiological changes and protection of different organs against ischaemic insults, potentially useful to treat dedicated patients.

Helium belongs to the family of noble gases, a group of chemical elements characterized by filled valence orbitals, carrying a maximum amount of electrons in the outer shell of the atom rendering them “inert” with very low chemical reactivity. Helium is the lightest noble gas with a molecular weight of 4 g/mol and has the lowest melting and boiling points of all elements [2]. In contrast to oxygen, helium has a lower density and viscosity, leading to reduced work of breathing; helium is therefore nowadays used in patients with obstructive airway diseases [2, 3].

On a cellular level several inert noble gases induce molecular changes leading to tissue protection in ischaemia-reperfusion situations [4]. Most studied today is xenon [5], which has been shown to have neuro- and cardioprotective effects [4, 6], although translation of animal data to humans does not always support protection in clinical settings [7–9]. However, xenon is rarely available and has anaesthetic properties, limiting application of higher concentrations to specific clinical situations. In contrast, helium is readily available and lacks anaesthetic properties; it is categorized as a “non-immobilizer”, a gas that is not able to induce anaesthesia (immobilize), but might having other behavioural effects. Helium has no haemodynamic side effects and it might therefore be useful for organ protection in critically ill patients. It has convincingly been shown that helium exerts cellular effects in vitro and in vivo: helium reduces ischaemia/reperfusion damage in cardiac [10, 11] and neuronal tissue [12–14]. A recent meta-analysis showed that helium has neuroprotective properties in rodents and is cardioprotective in rabbits [15]. For other organs like kidney and liver, there are still too few data to allow any conclusion whether helium has organ-protective effects.

This review will summarize and discuss the underling pathways of organ protection by helium, along with an updated review of current clinical uses of this inert gas in the critically ill patient.

## Conditioning organs against ischaemia

Ischaemic pre-conditioning, e.g. short periods of ischaemia interspersed with periods of reperfusion before a period of sustained ischaemia, was demonstrated in animals more than 20 years ago [16, 17] and has been described in most living cells and in all mammalian species. Conditioning the heart can be important in situations in which there is brief interruption of blood flow to an organ, e.g. during organ transplantation, vascular surgery, acute myocardial infarction and percutaneous coronary interventions (PCI), cardiac surgery with cardioplegic arrest, ischaemic stroke or post-cardiac arrest syndrome. Most intensively investigated is myocardial pre-conditioning, which refers to an application of short periods of ischaemia before a subsequent longer ischaemic period in the coronary vasculature. Preconditioning can be divided into an early (immediate effect, lasting for 2–3 h) and late phase of pre-conditioning (protection reappears after 24 h lasting for 2–4 days) [18]. Post-conditioning is achieved by application of short repetitive cycles of ischaemia within an organ/tissue directly at the onset of reperfusion [19, 20]. In contrast, remote pre-/per- or post-conditioning is defined by the application of sub-lethal periods of ischaemia to an organ distant from the target organ [21]. The obvious advantage of remote conditioning is that there is no need for additional manipulation of the threatened organ itself.

As applying ischaemia to an organ yields a high risk of complications for the patient, the search for alternative options to mimic ischaemic conditioning showed that different pharmacologic compounds activating adenosine, muscarinic, α-adrenergic, opioid or bradykinin receptors also contributed to organ protection [18]. Among other drugs and gases [22–24], helium is known to exert these organ-protective effects in animal models and partly also in human tissues [4, 25].

## What do we know about cardioprotection by helium (Table 1)

Already in 2007, it was shown that inhalation of three times 5 min of 70% helium before a subsequent coronary artery occlusion (30 min) and 3 h of reperfusion significantly reduced infarct size in rabbit hearts [26]. Using this approach, the authors employed several different inhibitors to block divergent signalling pathways in this helium induced cardioprotection, e.g. the so-called reperfusion injury salvage kinase (RISK) pathway. By using a phosphatidylinositol-3-kinase (PI3K) antagonist, a mitogen/extracellular signal-related kinase 1 (MEK-1) inhibitor and an inhibitor of the 70-kDa ribosomal protein s6 kinase (p70s6kinase) in an experimental in vivo set up, it was demonstrated that all three enzymes are causally involved in mediating the cardioprotection by helium [26]. The pro-survival kinases from the RISK pathway inhibited glycogen synthase kinase-3beta (GSK-3β) activity and activated apoptotic protein p53 degradation. By using a mitochondrial permeability transition pore (mPTP) opener (atractyloside), a crucial role of the mPTP in helium cardioprotection was established [27]. Subsequently, other key mediators like opioid receptors were identified to be involved in helium conditioning [28]. In addition, the non-selective nitric oxide synthase (NOS) inhibitor N-nitro-L-arginine methyl ester abolished cardioprotection by helium, while a selective inducible NOS inhibitor or a selective neuronal NOS inhibitor did not diminish helium-induced preconditioning [29].

**Table 1:** Enzymes and mediators involved in helium mediated cardioprotection

RISK pathway	
Phosphatidylinositol-3-kinase (PI3K)	
Mitogen/extracellular signal-related kinase 1 (MEK-1)	
70-kDa ribosomal protein s6 kinase (p70s6kinase)	
Inhibited glycogen synthase kinase-3beta (GSK-3β) activity	
Activated apoptotic protein p53 degradation	
Protein kinase A (PKA)	
Opioid receptors	
Endothelial nitric oxide synthase (NOS)	
Increased microparticle production in endothelial cells	
Reactive oxygen species (ROS)	
Maintaining intracellular acidosis during early reperfusion	
Cyclooxygenase 2 (COX2)	
Changes in ceramide	
Mitochondria	
Mitochondrial permeability transition pore (mPTP)	
Mitochondrial adenosine triphosphate-regulated potassium channel (mitoKATP)	
Uncoupling of mitochondrial respiration	
Mitochondrial calcium-sensitive potassium (mKCa) channel	
Caveolins	
Increased levels of circulating caveolin-3 in plasma	
Decreased levels of caveolin-1 and caveolin-3 in membrane fractions of myocardial cells	
Increased levels of caveolin-1 and caveolin-3 in ischaemic myocardium	

Known key players of ischaemic pre/post conditioning like the pro-survival kinases PI3K, ERK1/2 and their downstream targets: endothelial nitric oxide synthase (eNOS), p53 and GSK-3beta all converge at the mitochondrial level by preventing opening of the mPTP [30]. This is of critical importance as opening of the mPTP leads to mitochondrial dysfunction and finally cell death, and thus different preconditioning stimuli might preserve cardiac mitochondrial function by keeping the mPTP closed, thereby reducing tissue damage. Three critical mitochondria related mechanism have been identified to be involved in ischaemic cardioprotection: (1) opening of mitochondrial adenosine triphosphate-regulated potassium channel (mitoKATP), (2) generation of a small reactive oxygen species (ROS) burst and (3) maintenance of the mPTP. It is very likely that these mechanisms are also involved in preconditioning by noble gases and other pharmacological interventions. Helium-induced preconditioning has been shown to be mediated via preventing mPTP opening [31]: helium prevented mPTP opening by maintaining intracellular acidosis during early reperfusion. Additionally, the ROS scavengers N-Acetylcysteine and N-2-mercaptopropionyl glycine or the K-ATP-channel blocker 5-hydroxydecanoate abolished helium-induced preconditioning in rabbits, linking also the other two mentioned key pathways in the mitochondria to helium-induced cardioprotection [32]. This is supported by studies showing the involvement of mitochondria-related channels [33]: measuring cardiac mitochondrial function by determining the rate of oxygen consumption in isolated mitochondria, a direct effect of helium conditioning on mitochondrial function was demonstrated: helium induced a mild uncoupling of mitochondrial respiration [33]. Furthermore, the mitochondrial calcium-sensitive potassium (mKCa) channel blocker Iberotoxin abolished helium-induced infarct size reduction in these rats [33]. These data were confirmed in another study: the role of mKCa channels in helium induced cardioprotection was demonstrated by using NS1619, an *activator* of mKCa channels, which reduced the infarct size in both, young and aged animals. Moreover, the protein kinase A (PKA) blocker H-89 completely blocked this helium induced cardioprotection [34]. Looking more into detail of PKA regulation, it was found that the adenylyl cyclase activator forskolin reduced infarct size only in young animals. Thus, helium preconditioning is mediated by activation of PKA, and adaptions in PKA regulation might be an explanation for the age-dependent loss of cardioprotection by helium [34]. In a study using different concentrations of helium in a late preconditioning model, a helium concentration of 30% was still protective, while a lower concentration of 10% was not [35]. These data are of special interest for a clinical application of helium, as a low concentration of 30% of helium to achieve the threshold of protection allows the use of a significant amount of oxygen in critical ill patients. Helium was administered for 15 min 24 h before ischaemia/reperfusion, and cyclooxygenase 2 (COX2) was identified as a key mediator in helium late preconditioning [35]. Regarding postconditioning, prolonged helium application did not induce cardioprotection [36].

Employing gene expression screening methods, more details on the underlying mechanisms of helium-induced conditioning were revealed. Rats were subjected to either 5, 15, or 30 min of helium postconditioning, and a semi-quantitative histological analysis revealed that only 15 min of helium postconditioning decreased the ischaemia/reperfusion-induced cell damage [37]. With this 15 min helium postconditioning, 17 of 23 genes involved in necrosis, and 18 of 25 genes involved in pro-apoptosis were upregulated. Four of 23 (necrosis) and 7 of 25 genes (pro-apoptosis) were downregulated, and 9 of 11 anti-apoptotic genes as well as 24 of 32 genes involved in autophagy were upregulated after helium postconditioning. These data suggest that helium postconditioning alters dedicated targets in the cell death program [37]. Experimental studies demonstrated that conditioning is omitted or at least reduced by conditions such as diabetes, ageing and hypertension [38]. Only a few studies investigating helium conditioning in diseased animals have been performed. In healthy versus spontaneous hypertensive rats subjected to 25 min of myocardial ischaemia followed by 120 min reperfusion, 70% helium applied for 15 min after the index ischaemia (helium post-conditioning), combined with 15 min helium inhalation 24 h prior to index ischaemia (helium late pre-conditioning) or a triple intervention with additional 3 short cycles of 5 min helium inhalation shortly before ischaemia (helium early pre-conditioning) had inconsistent protective effects [39]. Only the triple intervention by pre-/post- and late conditioning was able to induce cardioprotection; a single intervention by helium postconditioning alone was however not protective. Molecular analyses demonstrated that helium conditioning was not associated with a mechanism involving GSK-3β and PKC-ε. Helium-induced early preconditioning and postconditioning were abolished in pre-diabetic obese Zucker rats in vivo [40]. Helium caused mild mitochondrial uncoupling in Zucker lean rats but not in Zucker obese rats. It was shown that helium had no effect on myocardial ERK1/2 and Akt phosphorylation in both animals’ types. However, GSK-3β phosphorylation was reduced in the heart only in Zucker lean animals but not in the obese rats [40].

## A novel target for helium conditioning: membrane proteins “caveolins”

All the aforementioned potential mechanisms probably involved in organ protection by helium leave one question unanswered: considering helium as an inert gas that would have a low activity to bind to specific receptors, thereby inducing signalling effects, an explanation for “how” helium would regulate differential protein expressions remained unknown. While it has been shown for xenon that van der Waals energy [41], charge-induced dipole interactions [42] and intermolecular complexes of xenon with small aromatic molecules play a role in cellular effects of this inert gas [19], similar findings for helium are yet not described.

We were intrigued by reports that gaseous anesthetics (volatile anaesthetics like, e.g. sevoflurane and isoflurane)-induced cardioprotection involves caveolins [43]. Membrane structures like caveolae and membrane proteins linked to these caveolae, the caveolins, might be important also in helium-induced organ protection [44–46].

Caveolins are structural proteins that are essential for the formation of caveolae. The role of caveolins in ischaemic and isoflurane-induced conditioning has been shown before [47–49]. Moreover, caveolins play a central role in several stress adaptation processes of different organs and tissues [50]. With their scaffolding domains caveolins can anchor and regulate cell proteins having been implicated in helium induced conditioning [51]. Among the three isoforms, caveolin-1 and caveolin-2 are expressed in numerous cell types (e.g. endothelial cells), while caveolin-3 is found primarily in skeletal and cardiac muscle, as well as in certain smooth muscle cells [52]. A recent review on caveolins summarizes the different cellular functions, e.g. as chaperones and scaffold recruiting signalling molecules [50]. As caveolins are considered to play a key role in the regulation of several multi-protein complexes [53], it is of special interest that many of the signalling molecules involved in cardiac protection by conditioning, including the G-alpha subunit of heterotrimeric G-proteins, Src kinases, PI_3_K, eNOS, PKC isoforms and ERK, are known to be regulated by caveolins [54, 55].

Studies aiming to elucidate in vivo a possible connection of helium to caveolins in fact showed that helium postconditioning increased levels of circulating caveolin-3 in plasma, along with increased levels of caveolin-1 and caveolin-3 in the myocardial area-at-risk (ischaemic area) in the rat heart in vivo [56, 57]. In a resuscitation model in rats, inhalation of helium (70%) for 5 min before cardiac arrest, plus 30 min after restoration of spontaneous circulation (ROSC) led to reduced myocardial apoptosis along with a differential expression of caveolin-1 and caveolin-3 in the heart [12]. We hypothesized that helium upregulates caveolin-1 and caveolin-3 in the myocardium, and therefore, mice inhaled 20 min either helium (70%)/oxygen (30%) or oxygen (30%)/air. Thirty minutes or 24 h after the experimental protocol, hearts were excised and blood was withdrawn from animals. In strong contrast to our initial hypothesis, the mice heart showed a significant downregulation of caveolin-1 and caveolin-3 after 24 h [58]. Subsequent sub-fractionation of the whole heart showed that specifically the membrane fractions, but not the cytosolic fractions, had decreased levels of caveolin-1 and caveolin-3. Corresponding to the decreased caveolin levels in the heart tissue and in the membrane fractions, increased levels of caveolin-3 in platelet free plasma of mice having inhaled helium were found [58]. This was confirmed by lowered cholesterol levels, a primary binding protein of caveolin in the plasma membrane. These data suggest that after helium inhalation caveolins might be secreted—together with lipids—from cardiac cell membranes into the blood stream. To confirm this, we mounted isolated mice hearts on a Langendorff system and repeated the respective experiments. The data showed that no helium protection was seen in this model lacking the in vivo blood flow [58]. It was further evaluated whether the secreted factors in the serum from helium-exposed mice have a measurable effect on mitochondrial function. The addition of helium conditioned serum to muscle cells in cell culture or naïve ventricular tissue increased mitochondrial metabolism without increasing ROS generation. Measurements of primary and lipid metabolites showed potential changes in ceramide in helium-conditioned serum [58]. Data from this mice study is in line with our previous findings in human endothelial cells investigating the direct effects of helium in vitro [59]. Administration of helium to endothelial cells in fact led to release of caveolins to the supernatant of these cells. In addition, the supernatant of the endothelial cells was able to preserve endothelial dysfunction by maintaining endothelial permeability [59].

**Fig. 1:**
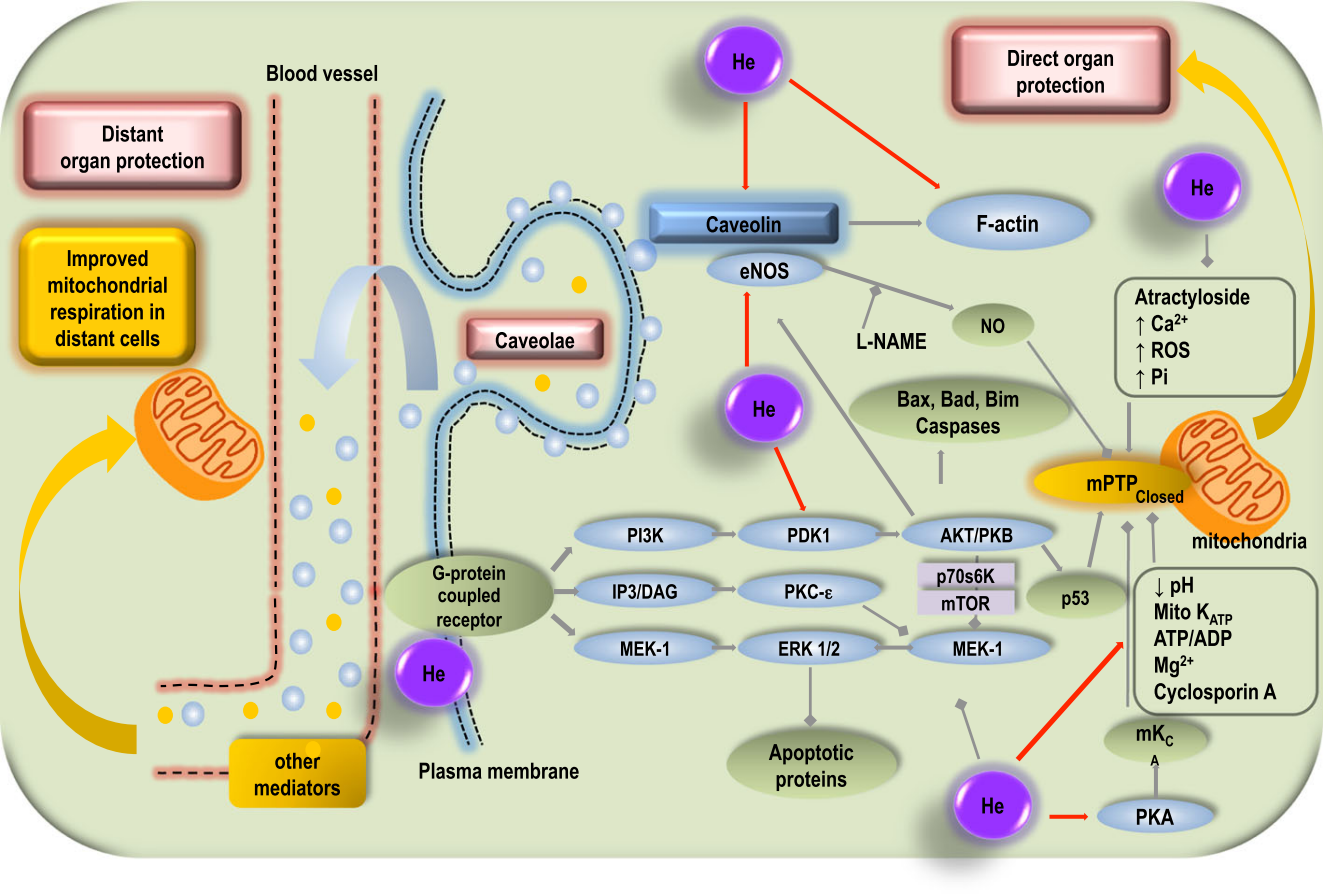
Mechanisms involved in helium-induced cardioprotection. This figure summarizes the known mechanisms in helium-induced cardioprotection, mainly via the RISK pathway, which is closely related to alterations in caveolin-related processes. Helium is depicted as a purple circle (He). Red arrows indicate an activating or upregulatory effect; squares indicate a suppressive or downregulatory effect. Intracellular the mechanisms converge on the mitochondria and preventing the mPTP from opening indicates cardioprotection. Also, the potential pathway of “remote condition” by helium has been depicted on the left side of the picture. Unknown and identified factors (caveolin, probably carried by exosomes) mediate a distant organ protection and can improve the mitochondrial respiration in distant cells. MEK-1, mitogen-activated protein kinase-extracellular signal-regulated kinase-1; ERK1/2, extracellular signal-regulated kinase 1/2; IP3, inositol triphosphate-3; DAG, diacylglycerol; PKC-ε, protein kinase C epsilon; GSK3β, glycogen synthase kinase 3β; PI3K, phosphatidylinositol-3-kinase, PDK-1, phosphoinositide-dependent protein kinase-1; PKB, protein kinase B; mTOR, mammalian target of rapamycin; P53, tumour protein P53; mPTP mitochondrial permeability transition pore; eNOS, endothelial nitric oxide synthase; NO, nitric oxide; L-NAME, L-NG-nitroarginine methyl esther; PKA, protein kinase A; mKCa, mitochondrial calcium-sensitive potassium channel; ROS, reactive oxygen species; Pi, inorganic phosphate; ATP, adenosine triphosphate; ADP, adenosine diphosphate

Regarding circulating factors mediating organ protection to remote organs, extracellular vesicle release has become of high interest [60]. As helium exposure can increase microparticle production in endothelial cells [61], we suggested that the production of microparticles is linked or even mediated via caveolin levels in the supernatant of cell cultures or in the blood stream and could in part be responsible for the protective effects of helium. These experimental findings are finally also in line with data from studies in human male volunteers [62]. Plasma was obtained from volunteers 6 h after helium inhalation. Cell damage was induced by hypoxia in isolated human endothelial cells in vitro. A 10% solution of plasma from volunteers previously exposed to helium decreased hypoxia induced cell damage. By caveolin-1 knockdown in the respective endothelial cells, a potential involvement of circulating caveolins was demonstrated [62]. We therefore concluded that helium induces the release of secreted membrane factors and microparticles that are enriched in caveolae/caveolins. All results taken together suggest a key role for these circulating factors in helium-induced organ protection. The experimental data for helium-induced conditioning are summarized in Fig. 1. Additional information on cellular changes mediated by caveolins and caveolae can be found elsewhere [50, 63, 64].

## Effects of helium in other organs

There are also studies investigating the effect of helium conditioning in other organs. Pretreatment with helium did not attenuate liver injury after warm ischaemia-reperfusion in the rat, although there was some evidence for modulation of the inflammatory response after hepatic ischaemia [65]. In contrast, Zhang et al. showed reduction of liver injury after helium preconditioning in mice, with the PI3K involved in the protective mechanism [66]. In human tubular kidney cells, helium in itself induced cell damage and increased cell damage after oxygen deprivation damage [67]. In contrast, Carmona et al. showed in dogs subjected to pneumoperitoneum that helium attenuates kidney damage if it was used for insufflation compared to carbon dioxide [68]. These data show that there is no constant protective effect of this noble gas in different organs, which holds also true for neuronal tissue. This is also supported by the meta-analysis performed by De Deken et al., showing that up to now, no conclusive data for helium application to prevent kidney or liver injury are available [15]. While we know that xenon is protective in the heart, neuronal tissue, kidney and other organs, it yet remains unsolved why helium has different effects in different organs.

Initially, neuroprotective properties of helium have been suggested in different brain damage models [42]. Post-ischemic neuroprotection after middle cerebral artery occlusion was caused by hypothermia induced by helium inhalation and not by direct cellular effects of the noble gas [69]. In a rat model of thromboembolic stroke, intra-ischemic helium at 75% inhibited tissue plasminogen activator-induced thrombolysis and subsequent reduction of ischemic brain damage, indicating that helium should not be given during thrombolysis [70]. However, post-ischemic helium at 75% reduced ischemic brain damage and brain haemorrhages and thus helium could be an efficient neuroprotective agent if given after tissue plasminogen activator-induced reperfusion [70]. In contrast, in neuronal cultures derived from foetal BALB/c mice cortices, which were damaged by oxygen and glucose deprivation (OGD), helium increased damage compared to control cells [47]. Pan et al. showed that hyperoxia (100% oxygen) had less effect on infarct volume reduction than had a mixture of heliox (30% oxygen/70% helium) during the ischemia/reperfusion process in the brain after focal ischaemia-reperfusion. Heliox improved neurological deficits at 24 h in these animal experiments [71]. This protection was only observed when heliox was administered immediately after cerebral artery occlusion: a 30–60 min delay abolished the infarct reducing effect [72]. Liu et al. examined the short and long-term neuroprotective effects of helium preconditioning in an neonatal cerebral hypoxia–ischemia model in rat pubs subjected to left common carotid artery ligation and then 90 min of hypoxia (8% oxygen at 37 °C) [13]. The authors showed that helium preconditioning reduced the infarct ratio, increased the number of survival neurons and inhibited apoptosis at the early stage of hypoxia-ischaemia insult. Furthermore, the sensorimotor function and the cognitive function were improved significantly in rats with helium preconditioning [13]. In another study from this group, helium preconditioning increased nitric oxide content, reduced the brain infarct area, increased anti-oxidases expression, decreased the number of apoptotic cells and improved neurological function and brain atrophy, an effect that was inhibited by a non-selective nitric oxide synthase inhibitor [73]. These data indicate that helium preconditioning attenuates hypoxia-ischaemia induced injury in the neonatal brain. Helium preconditioning ameliorated decompression-induced neurological deficits in rats [74].

However, other studies failed to show a neuroprotective effect of helium: inhalation of helium (50%) for 24 h did not improve histological or neurobehavioral assessment in rats subjected to 8 min of cardiac arrest [56]. In a model of neonatal asphyxia, argon, helium and xenon treatment to a different extend restored the cell morphology from mild ischemic/hypoxic insult. While argon and xenon treatment reduced infarct volume also from severe ischemia, helium had no protective effect [75]. Lack of neuroprotection for helium was recently confirmed: in contrast to xenon or argon, helium (75%) induced no protection against hypoxic-ischaemic injury in rat hippocampus in vitro [76]. Thus, data on neuroprotection are conflicting, and a final conclusion whether helium will be neuroprotective cannot be made at this moment.

## Is the organ protection by helium translatable to the clinical situation?

Although helium is used in patients with respiratory diseases, the enormous amount of data on helium induced organ protection is not yet translated to the clinical situation (Table 2). Failure to demonstrate translatability might include a number of reasons, e.g. age of the investigated population, concomitant diseases of patients, co-medication for concomitant diseases, differences in surgical trauma between standardized laboratory experiments and individual clinical situations, or just species differences.

**Table 2:** Effects of helium in ischemic human tissue

Release of secreted membrane factors and microparticles that are enriched in caveolae/caveolins	
Protecting human endothelium against ischaemia/reperfusion damage	
No changes in post-occlusive hyperaemic reactions in human endothelium	
Not any protective effect on post-operative troponin release after coronary artery bypass surgery	
No effect on the responsiveness of the human innate and early adaptive immune system	

There are up to date no clinical studies evaluating possible neuroprotective properties of helium. Regarding cardioprotection, patients undergoing coronary artery bypass grafting surgery were ventilated with a gas mixture containing helium (70%) for 3 × 5 min before start of cardiopulmonary bypass (helium pre-conditioning) or at the moment of coronary artery reperfusion after declamping of the aorta (helium post-conditioning) [77]. Surprisingly, neither helium pre- or postconditioning nor a combination of pre- and postconditioning had any protective effect on post-operative troponin release [77]. However, in healthy volunteers, helium protects human endothelium against ischaemia/reperfusion damage [78]. In a forearm blood flow model, endothelial function was measured by response to acetylcholine after 20 min of forearm ischaemia/reperfusion. Venous occlusion plethysmography measurement of the forearm blood flow in response to acetylcholine infusion showed that 3 × 5 min of 79% helium inhalation prevented post-ischaemic endothelial dysfunction. However, helium preconditioning did not affect plasma levels of cytokines, adhesion molecules, or microparticles. Also, a late endothelial preconditioning effect of helium could be shown 24 h after helium inhalation [78]. However, involvement of NOS, which has been previously demonstrated to be involved in experimental settings [29], could not be confirmed in humans. Another study in human healthy volunteers inhaling 50% helium before, during and after ischaemia used post-ischaemic reactive hyperaemia to determine endothelial function before and after 15 min of forearm ischaemia [79]. No changes in post-occlusive hyperaemic reactions were observed. An increase of the pro-inflammatory marker CD11b and ICAM-1 on leukocytes as well as an attenuated expression of the pro-coagulant markers CD42b and PSGL-1 on platelets were shown, suggesting a mild anti-inflammatory property of helium in humans [79]. Oei et al. investigated the effect of prolonged helium inhalation on the responsiveness of the human immune response in whole blood ex vivo [80]. Male healthy volunteers inhaled 30 min of heliox (79% He/21%O_2_) or air in a crossover design. Blood was withdrawn at different time points after helium inhalation and then incubated with lipopolysaccharide (LPS), lipoteichoic acid, T cell stimuli anti-CD3/anti-CD28 or pure media for 0, 2, 4 and 24 h. In this study, tumour necrosis factor-alpha (TNF-alpha), interleukin-1beta (IL-1beta), interleukin-6 (IL-6), interleukin-8 (IL-8), interferon-gamma and interleukin-2 (IL-2) were analysed by cytometric bead arrays. Helium inhalation did not influence the amounts of TNF-alpha, IL-1beta, IL-6, IL-8, IFN-gamma and IL-2 in comparison to air. Additionally, a group of volunteers inhaling helium for the extended period of 60 min did not show differences in cytokine production after LPS stimulation of whole blood [80]. Thus, this study did not show an effect of helium on the responsiveness of the human innate and early adaptive immune system.

## Ventilation improvement and lung-protective effects of helium

As a helium–oxygen mixture (heliox) reduces the work of breathing and gas trapping, heliox has been used long time for treatment of ventilation disorders, although very clear randomized controlled trials on this topic are still scarce. Acute respiratory stress can immediately be reduced by helium inhalation [81], as the low density of heliox renders turbulent airflow to laminar, subsequently lowering airway resistance and work of breathing. Heliox improved oxygen delivery during dynamic exercise in patients with chronic obstructive pulmonary disease (COPD) [82] and reduced dynamic lung hyperinflation, thereby improving indices of cardio-circulatory function like heart rate kinetics in the rest-to-exercise transition [83]. Heliox also reduced arterial carbon dioxide (CO_2_) tension; however, reduction of hypercapnia was mainly observed in spontaneously breathing and noninvasively ventilated helium-treated patients, but not in intubated patients during controlled ventilation [84]. These observations suggest that the decrease in arterial CO_2_ is based on reduced CO_2_ production, related to reduced work of breathing, and not improved alveolar ventilation. Improvement of pulmonary function test using helium as a driving gas for albuterol nebulization was only observed in patients with significantly reduced FEV_1_ [85].

A recent meta-analysis investigating the effects of non-invasive ventilation (NIV) with helium in patients with hypercapnic COPD exacerbation did not show a reduced rate for tracheal intubation in this patient population, although a lower incidence of NIV-related adverse events was observed [86]. A multicenter randomized controlled trial could also not show reduced intubation rates after heliox NIV, although heliox reduced the extend of respiratory acidosis and encephalopathy score [87]. It therefore remains questionable whether physiological improvement really leads to clinical benefits for the patient [88].

The lower density of heliox changes ventilation parameters during mechanical ventilation [89], and medical staff needs to be vigilant for possible ventilator disturbances [90]. Although several ventilators are available allowing heliox positive pressure ventilation, in the trial by Jolliet et al., heliox ventilation was stopped at the moment an endotracheal intubation was necessary [87]. Mechanical ventilation with helium has the potential to protect against ischaemic tissue damage after ischaemia-reperfusion has been occurred. In an explorative study, we showed that helium ventilation is feasible and can be safely used in patients treated with hypothermia after out-of-hospital cardiac arrest [91]. No adverse events related to the helium ventilation occurred during 3 h of ventilation with the noble gas. In animals, heliox improved CO_2_ removal during lung protective mechanical ventilation [92] and allowed for lower minute volume ventilation in subjects with ventilator-induced lung injury [93]. Helium inhalation after inducing adult respiratory distress syndrome in rats decreased neutrophil infiltration, interstitial/intraalveolar oedema, perivascular and/or intraalveolar haemorrhage and hyaline membrane formation [94]. Thus, next to non-invasive application of heliox, also mechanically ventilation of adults and children with heliox should be considered [95]. However, in mechanically ventilated subjects with severe air-flow obstruction, administration of heliox had no effect on indices of dynamic hyperinflation and resulted in only a small reduction in arterial CO_2_ tension [96]. Thus, it remains to be proven whether heliox inhalation really benefits severely pulmonary compromised patients [84, 88].

## Summary and conclusion

Though translation to the clinical practice of the protective effects of many conditioning agents failed, the current data show potential organ-protective effects of helium not only in animals but probably also in humans. Helium might therefore be of interest not only for patients with ventilation disorders. As helium has no anaesthetic side effects and no significant other off-target effects, it could be used in patients undergoing critical acute ischaemic events in numerous clinical situations. Whether application of helium in these clinical situations really reduces cell and organ damage, thereby improving patient outcome, needs further investigations.

## Data Availability

Not applicable
